# Supplementary material for: The Persistence of Antibiotic Resistance in Observational Studies: Is It Really Due to Differences in Sub-Populations Rather than Antibiotic Use?
Source: Antibiotics (Basel). 2025 Jan 6;14(1):39. doi: 10.3390/antibiotics14010039 (PMC11762111; doi:10.3390/antibiotics14010039)
Supplement: Supplementary file 1 [file antibiotics-14-00039-s001.zip › antibiotics-3364636-supplementary.pdf]

The Persistence of Antibiotic Resistance in Observational Studies: Is It Really Due to Differences in Sub-Populations Rather than Antibiotic Use?

Peter Collignon [1,2,] * and John J. Beggs [3]

1 Infectious Diseases Physician and Microbiologist, Canberra Hospital, Garran, ACT 0200, Australia
2 Medical School, Australian National University, Canberra, ACT 2601, Australia
3 Independent Researcher, Melbourne, VIC 3000, Australia; beggsjo_home@yahoo.com
* Correspondence: collignon.peter@gmail.com; Tel.: +61-2-6244-2891

Supplementary Material

S.1 Conditional Probability of Groups of Sequences of Outcomes

Denote the 27 prior clinical sequences as $\{S_j;\ 1,2,\ \ldots,27\}$. Let's denote the list of all prior clinical sequences that fall into a defined ith Group as G_i. Denote the proportion of the population in each community as C^A and C^B, and denote an Infected-Resistant outcome in period four by the symbol R_4. The conditional probability of being Infected-Resistant in fourth period given the individual comes from an historical sequence described in grouping G_i , $Prob(\ R_4|\ G_i\ )$, can be written:

$$Prob(\ R_4|\ G_i\ )\ = \frac{C^A \sum_{S_j \in G_i} Prob^A(S_j).Prob^A(R_4) +\ C^B \sum_{S_j \in G_i} Prob^B(S_j)\ .Prob^B(R_4)}{C^A \sum_{S_j \in G_i} Prob^A(S_j) +\ C^B \sum_{S_j \in G_i} Prob^B(S_j)}$$

where $\sum_{S_j \in G_i}()$ means to sum over all sequences in Group i.

S.2 Enumeration of and Probabilities of All Sequences

Outcome Sequences				Counters of Different Features of Outcomes in Each Sequence in the First Three Time Periods						Probability of Three Period Outcome Sequence in Each Sub-Population		Probability of Four Period Outcome Sequence in Each Sub-Population		Probability of the Fourth Period Outcome Given the Sequence in the First Three Periods
Period 1	Period 2	Period 3	Period 4	Looking from Period 4: Number of Time Periods Since Last Any Infection	Looking from Period 4: Number of Time Periods Since Last Resistant Infection	Number of Times Not Infected in First Three Periods	Number of Times Infected with a Antibiotic Sensitive Infection in First Three Periods	Number of Times Infected with a Antibiotic Resistant Infection in First Three Periods	Total Number of Times Infected First Three Periods	A: Prob Three Period Sequence	B: Prob Three Period Sequence	A: Prob Four Period Sequence	B: Prob Four Period Sequence	
N	N	N	N	4	4	3	0	0	0	0.17	0.61	0.09	0.52	0.819
N	I	N	N	2	4	2	1	0	1	0.06	0.07	0.03	0.06	0.771
I	N	N	N	3	4	2	1	0	1	0.06	0.07	0.03	0.06	0.771
I	I	N	N	2	4	1	2	0	2	0.02	0.01	0.01	0.01	0.692
N	R	N	N	2	2	2	0	1	1	0.08	0.04	0.04	0.03	0.708
R	N	N	N	3	3	2	0	1	1	0.08	0.04	0.04	0.03	0.708
I	R	N	N	2	2	1	1	1	2	0.03	0.00	0.02	0.00	0.630
R	I	N	N	2	3	1	1	1	2	0.03	0.00	0.02	0.00	0.630
R	R	N	N	2	2	1	0	2	2	0.03	0.00	0.02	0.00	0.588
N	N	I	N	1	4	2	1	0	1	0.06	0.07	0.03	0.06	0.771
N	I	I	N	1	4	1	2	0	2	0.02	0.01	0.01	0.01	0.692
I	N	I	N	1	4	1	2	0	2	0.02	0.01	0.01	0.01	0.692
I	I	I	N	1	4	0	3	0	3	0.01	0.00	0.00	0.00	0.618
N	R	I	N	1	2	1	1	1	2	0.03	0.00	0.02	0.00	0.630
R	N	I	N	1	3	1	1	1	2	0.03	0.00	0.02	0.00	0.630
I	R	I	N	1	2	0	2	1	3	0.01	0.00	0.01	0.00	0.581
R	I	I	N	1	3	0	2	1	3	0.01	0.00	0.01	0.00	0.581
R	R	I	N	1	2	0	1	2	3	0.01	0.00	0.01	0.00	0.563
N	N	R	N	1	1	2	0	1	1	0.08	0.04	0.04	0.03	0.708
N	I	R	N	1	1	1	1	1	2	0.03	0.00	0.02	0.00	0.630
I	N	R	N	1	1	1	1	1	2	0.03	0.00	0.02	0.00	0.630
I	I	R	N	1	1	0	2	1	3	0.01	0.00	0.01	0.00	0.581
N	R	R	N	1	1	1	0	2	2	0.03	0.00	0.02	0.00	0.588
R	N	R	N	1	1	1	0	2	2	0.03	0.00	0.02	0.00	0.588
I	R	R	N	1	1	0	1	2	3	0.01	0.00	0.01	0.00	0.563
R	I	R	N	1	1	0	1	2	3	0.01	0.00	0.01	0.00	0.563
R	R	R	N	1	1	0	0	3	3	0.02	0.00	0.01	0.00	0.555
N	N	N	I	4	4	3	0	0	0	0.17	0.61	0.03	0.06	0.110
N	I	N	I	2	4	2	1	0	1	0.06	0.07	0.01	0.01	0.126
I	N	N	I	3	4	2	1	0	1	0.06	0.07	0.01	0.01	0.126
I	I	N	I	2	4	1	2	0	2	0.02	0.01	0.00	0.00	0.153
N	R	N	I	2	2	2	0	1	1	0.08	0.04	0.02	0.00	0.147
R	N	N	I	3	3	2	0	1	1	0.08	0.04	0.02	0.00	0.147
I	R	N	I	2	2	1	1	1	2	0.03	0.00	0.01	0.00	0.173
R	I	N	I	2	3	1	1	1	2	0.03	0.00	0.01	0.00	0.173
R	R	N	I	2	2	1	0	2	2	0.03	0.00	0.01	0.00	0.187
N	N	I	I	1	4	2	1	0	1	0.06	0.07	0.01	0.01	0.126
N	I	I	I	1	4	1	2	0	2	0.02	0.01	0.00	0.00	0.153
I	N	I	I	1	4	1	2	0	2	0.02	0.01	0.00	0.00	0.153
I	I	I	I	1	4	0	3	0	3	0.01	0.00	0.00	0.00	0.177
N	R	I	I	1	2	1	1	1	2	0.03	0.00	0.01	0.00	0.173
R	N	I	I	1	3	1	1	1	2	0.03	0.00	0.01	0.00	0.173
I	R	I	I	1	2	0	2	1	3	0.01	0.00	0.00	0.00	0.190
R	I	I	I	1	3	0	2	1	3	0.01	0.00	0.00	0.00	0.190
R	R	I	I	1	2	0	1	2	3	0.01	0.00	0.00	0.00	0.196
N	N	R	I	1	1	2	0	1	1	0.08	0.04	0.02	0.00	0.147
N	I	R	I	1	1	1	1	1	2	0.03	0.00	0.01	0.00	0.173
I	N	R	I	1	1	1	1	1	2	0.03	0.00	0.01	0.00	0.173
I	I	R	I	1	1	0	2	1	3	0.01	0.00	0.00	0.00	0.190
N	R	R	I	1	1	1	0	2	2	0.03	0.00	0.01	0.00	0.187
R	N	R	I	1	1	1	0	2	2	0.03	0.00	0.01	0.00	0.187
I	R	R	I	1	1	0	1	2	3	0.01	0.00	0.00	0.00	0.196
R	I	R	I	1	1	0	1	2	3	0.01	0.00	0.00	0.00	0.196
R	R	R	I	1	1	0	0	3	3	0.02	0.00	0.00	0.00	0.198
N	N	N	R	4	4	3	0	0	0	0.17	0.61	0.04	0.03	0.071
N	I	N	R	2	4	2	1	0	1	0.06	0.07	0.02	0.00	0.103
I	N	N	R	3	4	2	1	0	1	0.06	0.07	0.02	0.00	0.103
I	I	N	R	2	4	1	2	0	2	0.02	0.01	0.01	0.00	0.155
N	R	N	R	2	2	2	0	1	1	0.08	0.04	0.02	0.00	0.145
R	N	N	R	3	3	2	0	1	1	0.08	0.04	0.02	0.00	0.145
I	R	N	R	2	2	1	1	1	2	0.03	0.00	0.01	0.00	0.197
R	I	N	R	2	3	1	1	1	2	0.03	0.00	0.01	0.00	0.197
R	R	N	R	2	2	1	0	2	2	0.03	0.00	0.01	0.00	0.225
N	N	I	R	1	4	2	1	0	1	0.06	0.07	0.02	0.00	0.103
N	I	I	R	1	4	1	2	0	2	0.02	0.01	0.01	0.00	0.155
I	N	I	R	1	4	1	2	0	2	0.02	0.01	0.01	0.00	0.155
I	I	I	R	1	4	0	3	0	3	0.01	0.00	0.00	0.00	0.205
N	R	I	R	1	2	1	1	1	2	0.03	0.00	0.01	0.00	0.197
R	N	I	R	1	3	1	1	1	2	0.03	0.00	0.01	0.00	0.197
I	R	I	R	1	2	0	2	1	3	0.01	0.00	0.00	0.00	0.229
R	I	I	R	1	3	0	2	1	3	0.01	0.00	0.00	0.00	0.229
R	R	I	R	1	2	0	1	2	3	0.01	0.00	0.00	0.00	0.241
N	N	R	R	1	1	2	0	1	1	0.08	0.04	0.02	0.00	0.145
N	I	R	R	1	1	1	1	1	2	0.03	0.00	0.01	0.00	0.197
I	N	R	R	1	1	1	1	1	2	0.03	0.00	0.01	0.00	0.197
I	I	R	R	1	1	0	2	1	3	0.01	0.00	0.00	0.00	0.229
N	R	R	R	1	1	1	0	2	2	0.03	0.00	0.01	0.00	0.225
R	N	R	R	1	1	1	0	2	2	0.03	0.00	0.01	0.00	0.225
I	R	R	R	1	1	0	1	2	3	0.01	0.00	0.00	0.00	0.241
R	I	R	R	1	1	0	1	2	3	0.01	0.00	0.00	0.00	0.241
R	R	R	R	1	1	0	0	3	3	0.02	0.00	0.00	0.00	0.246
